# A review on botanicals with wound healing activity for pemphigus vulgaris*:* perspective of traditional Persian medicine and conventional medicine

**Published:** 2017

**Authors:** Fatemeh Atarzadeh, Amir Mohammad Jaladat, Babak Daneshfard, Ladan Dastgheib, Mohammad Kamalinejad, Gholamreza Amin

**Affiliations:** 1 *Research Center for Traditional Medicine and History of Medicine, Shiraz University of Medical Sciences, Shiraz, Iran*; 2 *Essence of Parsiyan Wisdom Institute, * *Phytopharmaceutical Technology and Traditional Medicine Incubator* *, Shiraz University of Medical Sciences, Shiraz, Iran*; 3 *Department of Traditional Persian Medicine, School of Medicine, Shiraz University of Medical Sciences, Shiraz, Iran *; 4 *Research * *Center* * of Quran, Hadith and Medicine, Shiraz University of Medical Sciences, Shiraz, Iran*; 5 *Shiraz Molecular Dermatology Research Center, Department of Dermatology, Shiraz University of Medical Sciences, Shiraz, Iran *; 6 *School of Pharmacy, Shaheed Beheshti University of Medical Sciences, Tehran, Iran*; 7 *Department of Traditional Pharmacy, Tehran University of Medical Sciences, Tehran, Iran*

**Keywords:** Pemphigus vulgaris, Traditional Persian medicine, Herbal remedies

## Abstract

**Objective:**

As a rare autoimmune disease, pemphigus vulgaris has a poor prognosis especially in lack of proper medical support. This blistering disease involves both the skin and mucus membranes. The challenge is improving the healing process of skin lesions of which, superimposed infections are among the main causes of the disease mortality. Accordingly, we aimed to assess the treatment options suggested by traditional Persian medicine (TPM) and compare them with current findings.

**Materials and Methods:**

We studied the main clinical and pharmaceutical textbooks of TPM (Kitāb al-hāwīfī al-tibb, the Canon of Medicine, Eksir-e-Aazam, Tuhfat al-mu'minīn, Makhzan al-adviyah (focusing on the skin chapter and respective herbal remedies for the inflamed skin and ulcers. Additionally, scientific databases such as PubMed, Science direct, Scopus, and Google Scholar were searched for the current pharmacological evidence. In the studied books, the term “hot ulcers” was found close to what is known as “Pemphigus vulgaris”.

**Results:**

Reported medicinal herbs possess anti-inflammatory, antioxidant, wound healing, and antibacterial activities reported by recent studies. Therefore, they could be introduced as novel natural remedies for pemphigoid wounds.

**Conclusion:**

Taken as a whole, the review of traditional remedies for hot ulcers in Persian medical and pharmaceutical literature may open a new window toward developing new topical treatments for this disease.

## Introduction

Pemphigus vulgaris (PV) is a rare blistering autoimmune disease of the skin and mucus membranes. It is caused by auto-antibodies producedagainst antigens on the surface of keratinocytes (Kershenovich et al., 2014 ▶). There is no general agreement about the related treatment strategies. Treatment approaches mostly include establishment of disease remission in addition to effective control of it (Martin et al., 2011 ▶). These strategies are essentially conducted by systemic administration of corticosteroids. However, the undesirable side effects of these medicaments are often mentioned as the most frequent causes of morbidity and mortality (Poulin et al., 1984 ▶). Most important causes of death are: opportunistic superimposed infections, complications of long-term and high-dose usage of corticosteroids, and prolonged consumption of immune suppressant medicines (Ahmed, 2001 ▶).

Several investigations have been conducted on supplementary and natural medicaments to reduce the dose and consequently complications of the applied steroids (Ahmed, 2001 ▶; Mutasim et al., 2005 ▶; Poulin et al., 1984 ▶). Of course, it should be noticed that even such medicines may cause life-threatening infections as well as increased risk of infertility and cancer (Mutasim et al., 2005 ▶). Topical treatment approaches to PV are considered as supplementary therapy to systemic treatment. These strategies are believed to be effective via prevention of infection and promotion of re-epithelialization of the eroded skin (Ruocco et al., 2013 ▶).

In PV, skin lesions are among the most important sources of infection. In this regard, any topical treatment shortening the healing time of lesions and reducing the total drug dosage could be clinically reasonable (Tabrizi et al., 2007 ▶).

Herbal pharmacotherapy has a long history in traditional Persian medicine (TPM) (Golshani SA et al., 2015 ▶). With reference to the remaining documents of TPM which has been attributed to Unani medicine in Harrison's Principles of Internal Medicine (Kasper et al., 2015 ▶) and in line with our previous investigations (Atarzadeh et al., 2016 (a) ▶ and (b) ▶), the present study aimed at compiling a framework encompassing pharmacological and medical aspects of PV management in the light of TPM insights.

## Methodology

Initially, recent etiopathogenesis findings on PV emerged in PubMed, Scopus and Science direct databases were studied. The traditional term of “hot ulcer” was found very close to PV with regard to the clinical manifestations. Accordingly, description and etiological aspects of this disorder as well as pharmaceutical managements were derived from main medical and pharmaceutical manuscripts of TPM from the 10^th^ to 18^th^ centuries AD. The pharmaceutical literature included Tuhfat al-mu'minīn (MS P 21, 22- NLM, NLM Microfilm Reel: FILM 48-136 no. 2; The Present for the Faithful, written by Hakim MomenTonekaboni in 1670) and Makhzan al-adviyah (MS P 12- NLM, NLM Microfilm Reel: FILM 48-133 no. 2; The Storehouse of Medicaments, written by Aghili Shirazi 1670-1749 AD) (Shīrāzī, 2009 ▶; Tunakābunī, 2007 ▶). Medical literature was the Canon of Medicine by Avicenna (MS A 53, NLM, NLM Microfilm Reel: FILM 48-122 no. 5; 2^nd^ volume, 980-1037 AD) (4^th^ volume) and Eksir-e-Aazam (The Great Elixir written by Mohammad Azam Khan 1814-1902 AD) (Azam Khan, 2008 ▶; Ibn Sina, 1988 ▶).

Finally, to make a conclusion on collected medicaments, the results were compared and confirmed with the recent evidence on the herbs’ related pharmacological activities and therapeutic mechanisms of action.

**Etiologic factors involved in the pathogenesis of PV**

The main pathologic process in PV is “acantholysis” which is the result of cells detachment due to the destruction of their adhering glue, desmoglein by autoantibodies (Shah and Parmar, 2016 ▶). Although the cause of this autoimmune disease is originally unknown, some factors have been recognized to be involved in pathogenesis of the disease. For instance, there are circulating IgG autoantibodies directed against the normal desmogleins(Dsg) (a cell-to-cell adhesion molecule) (Kershenovich et al., 2014 ▶). T helper (Th) cells are also involved in the pathogenesis of PV. The role of auto-reactive T cells in induction and regulation of antibody production has been suggested in a recent study (Veldman et al., 2004 ▶). Moreover, contribution of interlukin-4 (IL-4), IL-5, IL-6 and IL-10 to the pathogenesis of PV suggests Th2 involvement (Okon and Werth, 2014 ▶). Oxidative stress is another issue which is considered to be involved in the etiology and pathogenesis of PV. In addition, there is a significant correlation between serum oxidative stress marker level and serum anti-desmoglein antibody level in PV (Abida et al., 2012 ▶).

**Traditional approach to ulcer**

“Wound” and “ulcer” are comprehensively outlined in the Canon of medicine with the special term of “jerahat” and “gharhe”. Ulcers may be hard or soft, hot or cold, septic or aseptic. Some have discharge but some do not, and finally they may be easily healed or may be difficult to treat. According to the TPM, “gharhe” is divided into hot and cold types. Cold ones have a broad base and white color, with minimal itching; in contrast, hot types are sharp with red base, itching, burning sensation, and irritation (Azam Khan, 2008 ▶; Ibn Sina, 1988 ▶). 

Pemphigus wounds are irritating with burning sensation, sometimes are itching, with prolonged healing time, and probably have superimposed infection (Mutasim et al., 2005 ▶). Although there may not be a distinct entity in TPM that could be fully attributed to PV, the aforesaid description seems to be relevant with hot ulcers in TPM sources. In other words, PV could be generally considered as a hot inflamed ulcer in TPM categorization of ulcers. Various natural topical remedies have been used in TPM for hot ulcer (Atarzadeh et al., 2016 (b) ▶). These remedies which have been used for many years by Iranian physicians are summarized in Table 1 (Azam Khan, 2008 ▶; Ibn Sina, 1988 ▶; Shīrāzī, 2009 ▶; Tunakābunī, 2007 ▶). In addition, *in vitro* and *in vivo* studies on plants used for hot ulcer healing in the studied ancient books are cited in Table 2.

## Discussion

Prolonged wound healing process of pemphigus erosions in addition to the associated pain, discomfort, and cosmetic problems are important determining factors in the duration of hospitalization and risk of secondary infections (Ali et al., 2006 ▶). Skin lesion is one of the most important sources of infection. For that reason, using local treatment, decreasing healing time of lesions, and reducing the total dosage of drugs are reasonable strategies (Tabrizi et al., 2007 ▶).

Clinical evidence about proper interventions for PV is scant and additional research is needed (Kasperkiewicz et al., 2012 ▶). Nowadays, Complementary and Alternative Medicine (CAM) has become more popular in different societies (Danish et al., 2011 ▶) and there is an increasing trend in its integration with healthcare systems. In this regard, our primogenitor has made a lot of discoveries about the healing effects of plants through trial and error (Jaladat et al., 2015 ▶) that could be helpful even inmodern-day applications.

**Table1:** Medicaments used for wound healing in hot ulcer and their underlying mechanisms of action.

**Scientific name**	**Traditional name**	**Part used**	**Applicatio** **n in Traditional medicine**	**Activities** ^*^											**Ref.**	
				**AO**	**AI**			**AB**	**WH**				**CT**			
***Portulacaoleracea*** ** L.**	*Baghlat-al-hamgha*	Aerial parts, Seed	Topical	**+**	**+**			**+**	**+**			**-**		(Alam et al., 2014; Kumar et al., 2008; Lee et al., 2012; Lim and Quah, 2007; Rashed et al., 2003)	
***Plantago ovata*** ** L.**	*Bazr-e-ghatouna*	Seed mucilage	Topical	**+**	**+**		**+**		**+**			**-**		(Masood and Miraftab, 2004; Motamedi et al., 2010; Rodríguez-Cabezas et al., 2003; Singh et al., 2011; Souri et al., 2008)	
***Rosa damascena*** ** Mill**	*Vard-e-ahmar*	Flower	Topical	**+**	**+**		**+**			**+**			**-**	(Boskabady et al., 2011; Hajhashemi et al., 2010; Kalim et al., 2010; Nikbakht and Kafi, 2004; Talib and Mahasneh, 2010)	
***Curcuma longa *** **L** ***.***	*Orogh-alsofr*	Root	Topical	**+**	**+**		**+**			**+**			**-**	(Chattopadhyay et al., 2004; Julie and Jurenka, 2009; Kim et al., 2001; Singh et al., 2002)			
***Plantago major *** **L.**	*Lesan al-hamal*	Seed mucilage	Topical	**+**		**+**		**+**			**+**		**-**	(Amini et al., 2010; Mahmood and Phipps, 2006; Reina et al., 2013; Sharifa et al., 2008)				

* AO: Antioxidant; AI: Anti-inflammatory; AB/AF: Antibacterial/Antifungal; WH: Wound healing; CT: Cytotoxicity

**Table2:** In vitro and in vivo studies on plants used in TPM for wound healing in hot ulcers.

**Ref.**	**Results** *	**Part used/** **Species**	**Scientific name**
(Rashed et al., 2003)	Wound healing	Fresh crude extract (Mice)	***Portulaca oleracea***
(Alam et al., 2014)	Antioxidant activity- ↓NO	Polysaccharide	
(Lee et al., 2012)	Anti-inflammatory, ↓TNF- α - ↓ROS- ↓MCP	Ethanol, aqueous extract	
(Yang et al. 2016; Agyare et al. 2015)	Antioxidant activity- Anti-inflammatory, ↓MPO, Il6, TNF- α	Ethanol extract	
(Motamedi et al., 2010)	Antimicrobial activity against Gram-positive and Gram-negative human pathogens	Methanol, ethanol extract	***Plantago ovata***
(Souri et al., 2008)	Antioxidant activity	Seed extract	
(Rodríguez-Cabezas et al., 2003)	Anti-inflammatory	Psyllium seeds (Rats)	
(Talib and Mahasneh, 2010)	Antibacterial activity against *Salmonella typhimurium*, *Bacillus cereus*, *Candida albicans* and Methicillin resistant *Staphylococcus aureus*	Butanol, aqueous extract	***Rosa damascena***
(Boskabady et al., 2011)	Antioxidant activity	Flower extract	
(Hajhashemi et al., 2010)	Anti-inflammatory, wound healing activity	Hydroalcoholic extract (Rats)	
(Fahimi et al., 2015)	wound healing activity	Oily extract of petals (Rats)	
(Chattopadhyay et al., 2004)	Anti- inflammatory-down regulation of cyclooxygenase-2. Significantly inhibit the generation ROS like superoxide anions, H2O2 and nitrite radical generation by activated macrophages, which play an important role in inflammation.	Curcumin	***Curcuma longa***
(Chattopadhyay et al., 2004)	significantly inhibit the generation ROS		
(Chattopadhyay et al., 2004)	Healing effect on both aseptic and septic wound	Turmeric powder (Rats, Rabbits)	
(Julie and Jurenka, 2009)	modulates the inflammatory response via inhibiting inflammatory cytokines production (TNF-α, IL-1,2,6,8, and 12, MCP)	Curcumin	
(Singh et al., 2002)	Antibacterial activity against pathogenic strains of Gram-positive and Gram-negative bacteria, Wound healing effect	Rhizome extracts	
(Chattopadhyay et al., 2004)	Reduce the TNF- α	Curcumin (Bovine)	
(Chattopadhyay et al., 2004)	Anti-inflammatory	Ethanol extract of rhizome, Curcumin, Sodium curcuminate (Rats)	
(Mahmood and Phipps, 2006)	Wound healing effect, Antioxidant activity	Aqueous extract	***Plantago major***
(Reina et al., 2013)	Anti-inflammatory activity	Hexane extract	
(Sharifa et al., 2008)	Antibacterial effects against Gram-positive and Gram-negative bacteria such as *Staphylococcus aureus* and *Escherichia coli*	Methanol extract	
(Reina et al., 2013)	↓ROS production	Baicalein and aucubin	
(Ameri et al., 2015)	Wound healing effect, Antioxidant activity, Anti-inflammatory activity		
(Akin et al., 2012)	Antibacterial activity on some Gram-positive and Gram- negative bacteria	Essential oil	***Myrtus communis***
(Aidi et al., 2010)	Antioxidant activity	Essential oil methanol extract, leaf, stem and flower	
(Choudhary et al., 2013)	Ability to modulate the immune response-↓NO - ↓T cell proliferation -↓ROS	Myrtucommuacetalone, myrtucommulone M, Myricetin	
(Aleksic and Knezevic, 2014)	Anti-inflammatory	Ethanol extract, Myrtucommulone (Rats, Mice)	
(Maxia et al., 2011)	Anti-inflammatory activity, ↓MPO activity- ↓TNF- α ↓IL6- ↓Leukocyte migration	Essential oil (Rats)	
(Asgarpanah and Ariamanesh, 2015)	Wound healing activity		
(Ravi et al., 2009)	Anti-inflammatory	Methanol extract	***Solanm nigrum***
(Kang et al., 2011)	↓NO-↓TNF- α - ↓IL6	Chloroform fraction	
(Sridhar et al., 2011)	Antibacterial activity against pathogenic bacteria such as *Bacillus subtilis*, *S. aureus*, *Klebsiella pneumonia*, *E. coli*	Ethanol, methanol, ethyl acetate, diethyl ether, leaves, seed and roots hexane and chloroform extract	
(Fahimi et al., 2015)	Wound healing activity	Aqueous extract of leaves(Rats)	
(Kibichiy et al., 2013)	Anti-inflammatory	Water extract (Mice)	
(Liu et al., 2016)	Antioxidant activity	Aqueous extract (Mice)	
(Parekh et al., 2005)	Antibacterial activity against *Staphylococcus aureus*, *B. cereus*	Methanol extract	***Santalum album***
(Ahmed et al., 2013)	Anti-inflammatory	HESP (Hydrolyzed Exhausted Sandal Wood Powder) oil (Rats)	
(Ahmed et al., 2013)	Antiulcer	Bark hydroalcoholic extract (Rats)	

* ROS: reactive oxygen species; IL: interleukin; MCP: monocyte chemoattractant protein; TNF-α: Tumor necrosis factor alpha; NO: Nitric oxide; MPO: myeloperoxidase

The therapeutic approach of Persian medicine for the treatment of ulcers is related to its specific classification for ulcers based on hotness and coldness, having discharge, extent, and rate of healing (Ibn Sina, 1988 ▶). Clinical features such as irritation, itching, red base, and burning sensation are somehow relevant with hot ulcers in TPM sources. Avicenna believed that these ulcers are sharp red-based ones which are accompanied by itching and irritation (Ibn Sina, 1988 ▶).

In TPM sources, many medicinal herbs have been mentioned for wound healing. Some of these herbs such as *Portulacaoleracea* L.,* Plantago ovata *Forssk. and* Plantago major* L. have mucilaginous characteristics and tissue regenerating ability andhave been used for hot swelling and hot ulcer topically. *Plantago major *L., for instance, could beapplied for removing the pus out of the infectedwound; thus,it is useful for burns and skin diseases*. Portulaca oleracea* L. and *Plantago ovata *Forssk. with rose oil are also beneficial for burns and hot ulcers (Shīrāzī, 2009 ▶).

*Plantago major *L. is a very effective remedy for superficial wounds. Its aqueous extract has shown wound healing effects in rats. Mechanism of action of its wound healing activity is related to its antioxidant properties (Mahmood and Phipps, 2006 ▶).

It has been suggested that treatment of white Swiss mice skin wounds with fresh homogenized crude extract of *Portulaca oleracea* has accelerated the wound healing process (Rashed et al., 2003 ▶).

*Plantago ovata *Forssk. Mucopolysaccharide also has beneficial properties for wound cleansing and wound healing (Masood and Miraftab, 2004 ▶).

It is to be mentioned that almost every herb proposed in TPM sources for hot ulcer treatment, has anti-inflammatory, antioxidant and wound-healing activities which have been shown to be beneficial in treatment of Pemphigus lesions, in recent studies. Moreover, they have antibacterial activity which could accelerate the wound healing process in infected wounds (Tables 1 and2). 

As the main limitation of our study, we only considered those herbal remedies with good levels of evidence reported by*in vivo* and/or *in vitro* wound healing investigations.

## Conclusion

In this review, we aimed at summarizing the approach of Persian medicine toward wound healing and particular herbs that can be used for Pemphigus ulcers re-epithelialization as a complementary treatment. 

In general terms, reviews on Persian medicinal herbs could pave the way for development of new herbal-based formulations for better management of Pemphigus lesions. While basic research provides data about wound-healing and the favorable effects of some herbs or herbal compounds, well-designed clinical trials should also be considered in the future.

## References

[B1] Abida O, Mansour RB, Gargouri B, Ayed MB, Masmoudi A, Turki H, Masmoudi H, Lassoued S (2012). Catalase and lipid peroxidation values in serum of Tunisian patients with pemphigus vulgaris and foliaceus. Biol Trace Elem Res.

[B2] Agyare C, Baiden E, Apenteng JA, Boakye Y, Adu-Amoah L (2015). Anti-infective and Anti-inflammatory Properties of Portulaca oleracea L. Don J Med Plant Res.

[B3] Ahmed AR (2001). Intravenous immunoglobulin therapy in the treatment of patients with pemphigus vulgaris unresponsive to conventional immunosuppressive treatment. J Am Acad Dermatol.

[B4] Ahmed N, Khan M, Jais AM, Mohtarrudin N, Ranjbar M, Amjad MS (2013). Antiulcer activity of sandalwood (Santalum album L) stem hydroalcoholic extract in three gastric-ulceration models of wistar rats. BolLatinoam Caribe Plant Med Aromat.

[B5] Akin M, Aktumsek A, Nostro A (2012). Antibacterial activity and composition of the essential oils of Eucalyptus camaldulensis Dehn and Myrtus communis L growing in Northern Cyprus. Afr J Biotechnol.

[B6] Alam MA, Juraimi AS, Rafii M, Abdul Hamid A, Aslani F, Hasan M, Mohd Zainudin MA, Uddin MK (2014). Evaluation of antioxidant compounds, antioxidant activities, and mineral composition of 13 collected purslane (Portulaca oleracea L) accessions. Biomed Res Int.

[B7] Aleksic V, Knezevic P (2014). Antimicrobial and antioxidative activity of extracts and essential oils of Myrtus communis L. Microbiol Res.

[B8] Ali A, AliReza Y, Gita F (2006). Pemphigus vulgaris in Iran: epidemiology and clinical profile. Skinmed.

[B9] Ameri A, Heydarirad G, Mahdavi Jafari J, Ghobadi A, Rezaeizadeh H, Choopani R (2015). Medicinal plants contain mucilage used in traditional Persian medicine (TPM). Pharm Biol.

[B10] Amini M, Kherad M, Mehrabani D, Azarpira N, Panjehshahin M, Tanideh N (2010). Effect of Plantago major on burn wound healing in rat. J Appl Anim Res.

[B11] Asgarpanah J, Ariamanesh A (2015). Phytochemistry and pharmacological properties of Myrtus communis L. Indian J Tradit Knowle.

[B12] Atarzadeh F, Daneshfard B, Dastgheib L, Jaladat AM, Amin G (2016 (a)). Early Description of Diet-Induced Blistering Skin Diseases in Medieval Persia: Avicenna's Point of View. Skinmed.

[B13] Atarzadeh F, Jaladat A, Dastgheib L, Amin G, Nimrouzi M, Kamalinejad M (2016 (b)). Cassia fistula: A remedy from Traditional Persian Medicine for treatment of cutaneous lesions of pemphigus vulgaris. Avicenna J Phytomed.

[B14] Azam Khan (2008).

[B15] Boskabady MH, Shafei MN, Saberi Z, Amini S (2011). Pharmacological effects of Rosa damascena. Iran J Basic Med Sci.

[B16] Chattopadhyay I, Biswas K, Bandyopadhyay U, Banerjee RK (2004). Turmeric and curcumin: Biological actions and medicinal applications. Curr Sci.

[B17] Choudhary MI, Khan N, Ahmad M, Yousuf S, Fun H-K, Soomro S, Asif M, Mesaik MA, Shaheen F (2013). New inhibitors of ROS generation and T-cell proliferation from Myrtus communis. Org Lett.

[B18] Danish M, Singh P, Mishra G, Srivastava S, Jha K, Khosa R (2011). Cassia fistula Linn (Amulthus)-An important medicinal plant: A review of its traditional uses, phytochemistry and pharmacological properties. J Nat Prod Plant Resour.

[B19] Fahimi S, Abdollahi M, Mortazavi SA, Hajimehdipoor H, Abdolghaffari AH, Rezvanfar MA (2015). Wound healing activity of a traditionally used poly herbal product in a burn wound model in rats. Iran Red Crescent Med J.

[B20] Golshani SA, Daneshfard B, Mosleh G, Salehi A (2015). Drugs and Pharmacology in the Islamic Middle Era. Pharm Hist (Lond).

[B21] Hajhashemi V, Ghannadi A, HajilooM (2010). Analgesic and anti-inflammatory effects of Rosa damascena hydroalcoholic extract and its essential oil in animal models. Iran J Pharm Res.

[B22] Ibn Sina (1988). Kitāb al-Qānūn fī al-ṭibb (Canon of medicine). Persian translation by Sharafkandi AR. Tehran.

[B23] Jaladat AM, Atarzadeh F, Rezaeizadeh H, Mofid B, Mosalaie A, Farhan F, Amin G (2015). Botanicals: An alternative remedy to radiotherapy-induced dysuria. Complement Ther Med.

[B24] Julie S, Jurenka MT (2009). Anti-inflammatory properties of curcumin, a major constituent. Altern Med Rev.

[B25] Kasper DL, Fauci AS, Hauser S, Longo D, Jameson JL, Loscalzo J (2015). Harrison's Principles of Internal Medicine.

[B26] Kalim MD, Bhattacharyya D, Banerjee A, Chattopadhyay S (2010). Oxidative DNA damage preventive activity and antioxidant potential of plants used in Unani system of medicine. BMC Complement Altern Med.

[B27] Kang H, Jeong H-D, Choi H-Y (2011). The chloroform fraction of Solanum nigrum suppresses nitric oxide and tumor necrosis factor-α in LPS-stimulated mouse peritoneal macrophages through inhibition of p38, JNK and ERK1/2. Am J Chin Med.

[B28] Kasperkiewicz M, Schmidt E, Zillikens D (2012). Current therapy of the pemphigus group. Clin Dermatol.

[B29] Kershenovich R, Hodak E, Mimouni D (2014). Diagnosis and classification of pemphigus and bullous pemphigoid. Autoimmun Rev.

[B30] Kim DS, Park SY, Kim JY (2001). Curcuminoids from Curcuma longa L (Zingiberaceae) that protect PC12 rat pheochromocytoma and normal human umbilical vein endothelial cells from βA (1–42) insult. Neurosci Lett.

[B31] Kibichiy SE, Jane M, Raymond M, Scolastica K, James K, John K (2013). Effects of crude extracts of Solanum nigrum on the Liver pathology and Survival time in Trypanosoma brucei rhodesiense infected mice. Sci J Microbiol.

[B32] Kumar BSA, Prabhakarn V, Lakshman K, Nandeesh R, Subramanyam P, Khan S, Ranganayakalu D, Krishna NV (2008). Pharmacognostical studies of Portulaca oleracea Linn. Rev Bras Farmacogn.

[B33] Lee AS, Kim JS, Lee YJ, Kang DG, Lee HS (2012). Anti-TNF-α activity of Portulaca oleracea in vascular endothelial cells. Int J Mol Sci.

[B34] Lim Y, Quah E (2007). Antioxidant properties of different cultivars of Portulaca oleracea. Food chem.

[B35] Liu F-P, Ma X, Li M-M, Li Z, Han Q, Li R (2016). Hepatoprotective effects of Solanum nigrum against ethanol-induced injury in primary hepatocytes and mice with analysis of glutathione S-transferase A1. J Chin Med Assoc.

[B36] Mahmood A, Phipps M (2006). Wound healing activities of Plantago major leaf extract in rats. Int J Trop Med.

[B37] Martin LK, Werth VP, Villaneuva EV, Murrell DF (2011). A systematic review of randomized controlled trials for pemphigus vulgaris and pemphigus foliaceus. J Am Acad Dermatol.

[B38] Masood R, Miraftab M (2004). Psyllium: current and future applications. Medical and Healthcare Textiles.

[B39] Maxia A, Frau MA, Falconieri D, Karchuli MS, Kasture S (2011). Essential oil of Myrtus communis inhibits inflammation in rats by reducing serum IL-6 and TNF-alpha. Nat Prod Commun.

[B40] Motamedi H, Darabpour E, Gholipour M, Nejad SS (2010). Antibacterial effect of ethanolic and methanolic extracts of Plantago ovata and Oliveria decumbens endemic in Iran against some pathogenic bacteria. Int J Pharmacol.

[B41] Mutasim DF, Bilic M, Hawayek LH, Pipitone MA, Sluzevich JC (2005). Immunobullous diseases. J Am Acad Dermatol.

[B42] Nikbakht A, Kafi M (2004). A study on the relationships between Iranian people and Damask rose (Rosa damascena) and its therapeutic and healing properties. In VIII International People-Plant Symposium on Exploring Therapeutic Powers of Flowers.

[B43] Okon L, Werth V (2014). Bullous Pemphigoid, Mucous Membrane Pemphigoid and Pemphigus Vulgaris: An Update on Pathobiology. Curr Oral Health Rep.

[B44] Parekh J, Jadeja D, Chanda S (2005). Efficacy of Aqueous and Methanol Extracts of Some Medicinal Plants for Potential Antibacterial Activity. Turk J Biol.

[B45] Poulin Y, Perry HO, Muller SA (1984). Pemphigus vulgaris: results of treatment with gold as a steroid-sparing agent in a series of thirteen patients. J Am Acad Dermatol.

[B46] Rashed A, Afifi F, Disi A (2003). Simple evaluation of the wound healing activity of a crude extract of Portulaca oleracea L(growing in Jordan) in Mus musculus JVI-1. J Ethnopharmacol.

[B47] Ravi V, Saleem TM, Patel S, Raamamurthy J, Gauthaman K (2009). Anti-inflammatory effect of methanolic extract of Solanum nigrum Linn berries. Int j appl res nat prod.

[B48] Reina E, Al-Shibani N, Allam E, Gregson KS, Kowolik M, Windsor LJ (2013). The effects of plantago major on the activation of the neutrophil respiratory burst. J Tradit Complement Med.

[B49] Rodríguez-Cabezas M, Galvez J, Camuesco D, Lorente M, Concha A, Martinez-Augustin O, Redondo L, Zarzuelo A (2003). Intestinal anti-inflammatory activity of dietary fiber (Plantago ovata seeds) in HLA-B27 transgenic rats. Clin Nutr.

[B50] Ruocco E, Wolf R, Ruocco V, Brunetti G, Romano F, Schiavo AL (2013). Pemphigus: associations and management guidelines: facts and controversies. Clin Dermatol.

[B51] Shah J, Parmar D (2016). Pemphigus: Complete Literature on Symptoms, Pathogenesis & Treatment. Indian J Appl Res.

[B52] Sharifa A, Neoh Y, Iswadi M, Khairul O, Abdul Halim M, Jamaludin M, Mohamed Azman A, Hing H (2008). Effects of methanol, ethanol and aqueous extract of Plantago major on gram positive bacteria, gram negative bacteria and yeast. Ann Microsc.

[B53] Shīrāzī MA (2009). Makhzan al-adviyah (The Storehouse of Medicaments).

[B54] Singh R, Chandra R, Bose M, Luthra PM (2002). Antibacterial activity of Curcuma longa rhizome extract on pathogenic bacteria. Curr Sci-Bangalore.

[B55] Singh S, Singh R, Kumar N, Kumar R (2011). Wound healing activity of ethanolic extract of Plantago Ovata (Ispaghula) seeds. Rev ciênc farm básica apl.

[B56] SouriE, Amin G, Farsam H (2008). Screening of antioxidant activity and phenolic content of 24 medicinal plant extracts. Daru.

[B57] Sridhar T, Josthna P, Naidu C (2011). In vitro antibacterial activity and phytochemical analysis of Solanum nigrum (Linn)-An important antiulcer medicinal plant. J Exp Sci.

[B58] Tabrizi M, Chams‐Davatchi C, Esmaeeli N, Noormohammadpoor P, Safar F, Etemadzadeh H, Ettehadi H, Gorouhi F (2007). Accelerating effects of epidermal growth factor on skin lesions of pemphigus vulgaris: a double‐blind, randomized, controlled trial. J Eur Acad Dermatol Venereol.

[B59] Talib WH, Mahasneh AM (2010). Antimicrobial, cytotoxicity and phytochemical screening of Jordanian plants used in traditional medicine. Molecules.

[B60] Tunakābunī MD (2007). Tuhfat al-mu’minīn (The Present for the Faithful). Research center of traditional medicine. Shahid Beheshti university of medical sciences.

[B61] Veldman C, HöhneA, Dieckmann D, Schuler G, Hertl M (2004). Type I regulatory T cells specific for desmoglein 3 are more frequently detected in healthy individuals than in patients with pemphigus vulgaris. J Immunol.

[B62] Wannes WA, Mhamdi B, Sriti J, Jemia MB, Ouchikh O, Hamdaoui G, Kchouk ME, Marzouk B (2010). Antioxidant activities of the essential oils and methanol extracts from myrtle (Myrtus communis var italica L) leaf, stem and flower. Food Chem Toxicol.

[B63] Yang X, Yan Y, Li J, Tang Z, Sun J, Zhang H (2016). Protective effects of ethanol extract from Portulaca oleracea L on dextran sulphate sodium-induced mice ulcerative colitis involving anti-inflammatory and antioxidant. Am J Transl Res.

